# Measuring patient activation: the utility of the Patient Activation Measure administered in an interview setting

**DOI:** 10.1007/s11136-024-03614-2

**Published:** 2024-02-22

**Authors:** Magdalena Holter, Alexander Avian, Martin Weger, Sanja Strini, Monja Michelitsch, Katja Brenk-Franz, Andreas Wedrich, Andrea Berghold

**Affiliations:** 1 Institute for Medical Informatics, Statistics and Documentation, Medical University of Graz, Graz, Austria; 2 Department of Ophthalmology, Medical University of Graz, Graz, Austria; 3 Institute of Psychosocial Medicine, Psychotherapy and Psychooncology, Jena University Hospital, Jena, Germany

**Keywords:** Patient Activation Measure®, Psychometrics, Item response theory, Mode of questionnaire administration

## Abstract

**Background:**

Patient activation is an emerging field in healthcare research concerning knowledge, skills, and confidence of patients in managing their health. This is particularly important for patients with chronic diseases, who often require more complex care management and self-care skills. However, due to temporary or longer-lasting visual impairments, certain patient groups cannot answer a questionnaire independently. The main objective is to investigate the psychometric properties of the German Patient Activation Measure® (PAM) survey in an everyday clinical setting where it has to be read aloud.

**Methods:**

Outpatients with macular edema participated in this questionnaire-based cross-sectional study. The study assessed patient activation by the PAM® survey, self-rated health, self-efficacy, quality of life, and general mood. Interviewers read questionnaires aloud to patients. Psychometric properties of the PAM® survey were investigated by item response theory (IRT), Cronbach’s *α* and trait–trait correlations.

**Results:**

The analysis included *N* = 554 patients. Median age was 69 (IQR 62.0–76.0) years and mean overall activation score 74.1 (SD 13.7). All items showed ceiling effects. Empirical reliability from the IRT model and Cronbach’s *α* were 0.75. The PAM® survey showed a Spearman correlation of 0.54 with self-efficacy, 0.51 with quality of life and 0.34 with general mood.

**Conclusion:**

The read-aloud PAM® survey has been shown to provide to adequate measurement precision and convergent validity to be used as a screening tool in an everyday clinical setting. Objective assessment in an interview setting with the PAM® survey is possible. PAM® items are good in distinguishing lower to middle activated patients, but not patients with high activation. Further, issues with structural validity need more investigation.

**Supplementary Information:**

The online version contains supplementary material available at 10.1007/s11136-024-03614-2.

## Plain English Summary

Patient activation, the patients' knowledge, skills, and confidence in managing their disease is crucial for better health outcomes. The Patient Activation Measure (PAM®) is used in different healthcare settings to gain insight about patient’s activation level. However, it is unclear if the PAM® works as intended with patients suffering from a chronic disease affecting the eye, unable to read and fill the questionnaire by themselves. In everyday clinical settings, we used the PAM® survey with patients suffering from macular edema, ensuring accessibility by reading it aloud. This study indicates that objective assessment in an interview setting with the PAM survey is possible. The PAM® survey showed to sufficiently measure patient activation if read aloud. It can be made accessible to many patients as it can be used in interviews. The PAM® survey quickly identifies patients with low patient activation.

## Background

The number of adult patients suffering from a chronic disease e.g. diabetes or hypertension is steadily rising worldwide [1]. Those patients have to deal with complex treatments, may have to change their lifestyle and should be aware when their health status may deteriorate. To provide a better health outcome, patients suffering from a chronic disease should engage in their health care [2]. Therefore, patient activation is essential [2]. It describes knowledge, skills, and confidence of patients in managing their own health. More activated adult patients use more self-management services, show better self-management behavior, and are more compliant in taking their medication [3]. In addition, they believe in being in control of their own health [4]. Highly activated patients show more self-efficacy [5–7], higher quality of life [3, 8, 9] and lower depression scores [10, 11]. Patient activation can be increased through interventions, such as patient education or self-management coaching [12].

Patient activation can be assessed by the patient activation measure (PAM® survey), a widely used self-report questionnaire [3, 4, 6–9, 13]. It consists of 13 statements regarding ones’ health behavior and attitudes towards health management. By the PAM® survey, a patient can be categorized into one of four levels of patient activation. At level 1, patients are not engaged in managing their own health; at level 2, they lack confidence to take action to maintain and improve their health; at level 3, they take action by positive changes in their health behaviors; and at level 4, they maintain a healthy lifestyle even during stressful times. Reliability and validity of the questionnaire were endorsed by multiple studies e.g. [2, 13]. The PAM® survey was translated into several languages [14, 15] including German [5] and completed by various patient groups (e.g., mental problems, neurological diseases, multimorbid patients [13]). Patients with wet age-related macular degeneration showed high activation [16]. The activation scores of patients with vascular diseases were in a normal range [17]. For patients with diabetes slightly lower scores were found [18, 19]. Overall, the PAM® survey seems to be a reliable and valid measurement tool for assessing patient activation in multiple languages and different patient groups.

Nonetheless some patient groups are not able to complete patient-reported outcomes on their own, e.g., because of impaired vision. For those patient’s questionnaire can be made accessible in an interview setting. However, since most questionnaire are developed for self-administration, completing them in an interview could potentially lead to different results [20]. The self-administered paper questionnaire is visual, while the interview setting is aural, which can evoke different cognitive processes and therefore different responses [20]. Moreover, the interaction between the interviewer and participant can vary and the issue of interviewer bias arises [21]. This describes the unconsciously reaction of an interviewer in a way that prompts the participant to provide a response that is biased toward the interviewer's reactions [21]. Additionally, during interviews participants provided more positive and socially acceptable answers than during self-administration [20].

The PAM® survey was self-administered and applied in an interview setting [22]. From a psychometric perspective it is of interest how the PAM® survey works in case it is read aloud, which is especially relevant for patients with a chronic disease affecting vision and not known yet. The main goal of this study was to gain insight into how the PAM® survey can be used in an everyday clinical setting where it needs to be read aloud for patients.

## Methods

### Study design

This is a questionnaire-based cross-sectional study. It consists of an ad hoc sample from the population of outpatients from the Department of Ophthalmology of the Medical University of Graz. Data collection was performed from March 2020 until the end of February 2022. The ethical committee of the Medical University of Graz approved the study (32-101 ex 19/20).

### Participants

A macular edema is an accumulation of fluid in the macula impairing vision and can lead to severe visual impairment. It is a heterogeneous disease with different possible etiologies. This study focuses on two different etiologies: diabetes and retinal vein occlusion, the most common retinal vascular diseases [23]. Diabetic macular edema represents the most prevalent subtype, affecting an estimated 5.5% of the global diabetic population [24]. While diabetic macular edema develops slowly and occurs mostly bilateral, the onset of retinal vein occlusion is sudden and most frequently only one eye is affected. Most patients with a macular edema are treated with intravitreal injections, which means monthly visits at the hospital. Macular edemas due to diabetes and retinal vein occlusion can also be treated by patients’ health behavior. Both types of macular edema have similar risk factors and are due to vascular risk factors.

This study enrolled patients with center-involving macular edema resulting from diabetes or retinal vein occlusion. They had to be aged 18 or older, speak German well enough for questionnaire comprehension, and possess hearing abilities for verbal communication. Exclusion criteria involved cognitive impairment.

### Data collection

After signing informed consent, each patient enrolled was asked to complete the questionnaires once. This took place in-between medical routine eye-examinations. Because of these examinations patients could not read questionnaires by themselves. Thus, questionnaires were read out loud by one of four trained interviewers (MH, VW, AK, JG) using a standard procedure to guarantee objectivity and comparability of each interview. The training included practicing the interview procedure and familiarizing with outcome measures. Possible barriers and difficulties encountered during interviews were discussed, and appropriate behavior rehearsed. For instance, guiding patients to respond using a scale rather than providing a narrative or story was practiced, without influencing their answers towards any particular category. Possible answers were printed out in big font and located in front of patients. Participants were pseudonymized with the web-based pseudonymization tool ‘iPSN’ [25]. LimeSurvey [26] was used to gather and store answers of participants with their pseudonym.

### Outcome measures

The PAM® survey [13] assesses knowledge, skills, and confidence of patients in managing their own health. The items are rated on a Likert-type scale with five response categories from “Disagree strongly” to “Agree strongly” and “Not applicable”. Answers to the 13 items are summed up and transformed to a scale between 0 and 100 [13]. The German version of the PAM® survey (PAM-13D) [5] has been a reliable and valid questionnaire, showing a Cronbach’s *α* of 0.84, factorial structure and a trait–trait correlation of *r* = 0.43 between the score of the PAM-13D and general self-efficacy [5].

Furthermore, the trait well-being inventory mood level scale [27] was used to measure general mood and general quality of life. Cronbach’s *α* is 0.83 for the scale general mood and 0.87 for the scale general quality of life [27]. Quality of life was conceptualized as an overall measure of life satisfaction, focusing on the cognitive dimension of subjective well-being encompassing beliefs about the present, past, and future, rather than specific aspects of life satisfaction in distinct domains. Strong positive correlations with life-satisfaction indicated construct validity [27]. Moreover, we assessed subjective belief to successfully cope with new demanding situations by own strength by the general self-efficacy scale [28]. In several German samples, a Cronbach’s *α* between 0.80 and 0.90 was found [28]. Validity is given by correlations of self-efficacy with various other constructs, such as negative correlations with depression, anxiety, and burnout. For these questionnaires used in the study, mean score over item answers were built. Additionally, self-perceived health status was rated in five categories (“Very bad”, “Bad”, “Moderate”, “Good”, “Very good”) [29]. Moreover, demographic data like sex, age, and education were assessed. Net income was investigated in five categories, corresponding to the income quintiles for elderly in Austria (www.statistik.at).

### Data analysis

To achieve the study objective of investigating the psychometric properties of the PAM, a minimum of 500 patients were required to obtain stable estimates [30], further information are included in the Supplementary Files.

Categorical data are presented as absolute and relative frequencies, continuous data as means and standard deviations or medians and interquartile ranges, as appropriate. To gain the PAM® score, the answer category “Not applicable” was transformed into missing values and the raw scores of the PAM-13D were summed up and transferred to a scale between 0 and 100, according to the algorithm by Insignia Health, the company licensing the questionnaire (https://www.insigniahealth.com/products/pam).

To analyze the PAM-13D, we used item response theory (IRT), a set of statistical models describing the relationship between questionnaire items and person ability. Ability is observed through the answers given to questionnaire items. With higher person ability, higher categories are chosen (e.g., “Agree strongly”). Firstly, assumptions of IRT analysis were examined. The generalized partial credit model (GPCM) was chosen out of different IRT models based on fit-indices, LR-tests and Vuong tests (see Supplementary Table 1). This model estimates two different parameters for each item: item difficulty and item discrimination. Item difficulty describes how easy persons agree with the item. Item discrimination describes how well an item distinguishes persons with high and low ability.

To evaluate model fit, we used root mean square error of approximation (RMSEA), standardized root mean square residual (SRMSR), Tucker–Lewis index (TLI), comparative fit index (CFI). Good fit was defined as a RMSEA < 0.05, SRMSR ≤ 0.08 and > 0.9 for TLI and CFI [31]. Sample adjusted Bayesian information criterion (SABIC) and Akaike information criterion corrected (AIC_c_) were examined as well, smaller values indicate a better model fit. We used infit and outfit statistics to evaluate item to model fit. The range of 0.5–1.5 is efficient for measurement, while the area between − 1.9 and 1.9 describes reasonable predictability. Values ≤  − 2 indicate data are too predictable [32]. Moreover, the relationship of choosing between answer categories of items and ability of patients is shown in a wright map. We used a test information curve to show the amount of ability measured over the ability range and to estimate the standard error. The GPCM was used to calculate the number of empirical distinguishable groups by the separation index [33]. To ensure answer behavior was not influenced by interviewers, differential item functioning (DIF) was assessed. The likelihood-ratio *χ*^2^ test was used to detect DIF. McFadden’s pseudo-*R*^2^ and non-compensatory differential item functioning (NCDIF) were used as a measure of DIF magnitude.

Furthermore, floor and ceiling effects were defined as > 15% of answers in the lowest or highest answer category, respectively [34]. Additionally, item difficulty was investigated according to classical test theory (CTT), represented as item mean. For assessing CTT reliability, we used Cronbach’s *α* (inner consistency). A value above 0.7 indicates acceptable reliability [35]. Indications for construct validity were gained through trait–trait correlations between the PAM® score and other questionnaires. It was expected that patient activation would be moderately negatively associated with self-related health status [36–38], moderately positively associated with self-efficacy [5], quality of life [9, 39, 40], and general mood [9, 41, 42], and weakly correlated with perceived social support [42, 43]. Correlations coefficients were judged as small if > 0.10, as medium if > 0.30 and as large if > 0.50 [44]. Group differences were evaluated by means of an Analysis of Variance. In case the overall comparison was significant, Tukey's HSD test was used for specific group differences.

Statistical analysis was performed using R studio version 4.1.1 [45] using the packages mirt [46] and lordif [47].

## Results

### Study participants

Overall, 707 patients were screened for eligibility. 81 Individuals (11%) were deemed ineligible due to inadequate German proficiency, cognitive incapacity, auditory impairments, or frailty 39 (6%) declined study participation. Six (1%) withdrew informed consent before the interview was started. Twelve interviews (2%) had to be stopped because patients were not suitable (e.g., hearing difficulties); 5 patients (1%) did the interview twice. 10 (1%) Interviews were incomplete, so 554 (78%) patients’ data sets were available for analysis. *N* = 321 (58%) of patients were male and median age was 69 (IQR 62–76). 317 (57%) Patients suffered from a macular edema due to diabetes, 224 (40%) due to retinal vein occlusion and 13 (2%) exhibited both types (see Table 1).

**Table 1:** PAM® scores by socio-demographic and health characteristics (*n* = 554)

	Total	PAM*®* score	*p*-value^b^
Age (years)	69.0 (62.0 –76.0)		
Sex			0.018
Male	321 (58%)	72.9 ± 13.4	
Female	233 (42%)	75.7 ± 13.9	
Education			0.045^c^
Basic education	411 (74%)	74.9 ± 13.8	
High school	68 (12%)	71.1 ± 14.0	
Higher education	75 (14%)	72.2 ± 12.3	
Working status			0.041^d^
Working	66 (12%)	73.5 ± 12.7	
Other^a^	41 (7%)	69.1 ± 13.8	
Retired	447 (81%)	74.6 ± 13.7	
Monthly net income			0.172
< 800€	72 (13%)	75.2 ± 13.1	
< 1125€	91 (17%)	71.7 ± 14.5	
< 1500€	112 (21%)	76.3 ± 13.7	
≤ 1950€	82 (15%)	74.0 ± 12.7	
> 1950€	184 (34%)	73.5 ± 13.5	
Missing	13 (2%)	74.4 ± 18.1	
BMI	25.8 (24.7–30.0)		
Type of macular edema	0.327		
Diabetic	317 (57%)	73.4 ± 13.7	
Retinal vein occlusion	224 (40%)	75.1 ± 13.7	
Both	13 (2%)	72.0 ± 11.3	
Comorbidity			0.224
Diabetes	129 (23%)	74.6 ± 12.6	
Hypertension	196 (35%)	75.1 ± 13.5	
Both	229 (41%)	72.9 ± 14.4	
Health status self-rated			< .001^e^
Very good	61 (11%)	83.3 ± 11.1	
Good	236 (43%)	76.6 ± 11.7	
Moderate	222 (40%)	70.6 ± 13.8	
Bad	29 (5%)	60.8 ± 14.1	
Very bad	6 (1%)	72.7 ± 15.4	
Visual acuity tested			
Both eyes	350 (63%)		
Only worse eye	160 (29%)		
No data	44 (8%)		

Visual acuity		LogMAR
Better eye	350	0.19 ± 0.20
Worse eye	510	0.42 ± 0.36

Data are presented as *N* (%), mean ± SD or median (25th–75th percentiles)

*BMI* body mass index

^a^Category ‘Other’ includes jobless, studying and homemaker

^b^Estimated difference in the PAM® score by an Analysis of Variance

^c^No significant difference between any specific groups in the post-hoc test (Tukey's HSD)

^d^Post hoc test retired vs. other *p* = 0.032

^e^Post hoc test *p* < 0.01 for very good vs. good; *p* < 0.001 for very good vs. moderate and bad, good vs. moderate and bad, moderate vs. bad. No missing data in PAM® scores

There were four different interviewers with interviewer MH doing 185 (33%), interviewer VW 159 (29%), interviewer AK 114 (21%) and interviewer JG 96 (17%) interviews, where each interviewer conducted as many interviews as feasible. One interview took about 20 min (IQR 16.4–24.8). The PAM-13D was finished in approximately four minutes (IQR 2.7–4.7).

### PAM-13D results

**Fig. 1:**
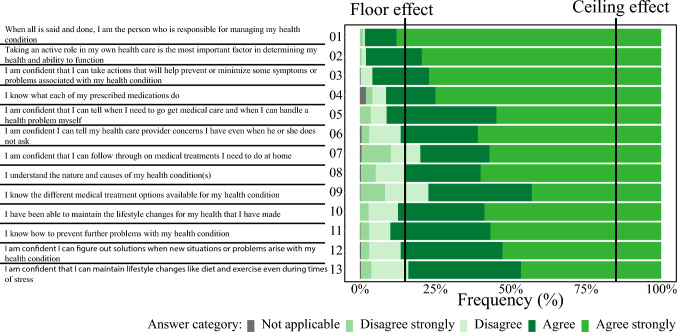
Category answer frequencies for the PAM®

The response “Not applicable” was chosen most frequently for item 4, occurring in 2% of all cases. The most frequent response category across all items was “Agree strongly”, which was selected between 43% (item 9) and 88% (item 1) of patients, see Fig. 1. 10–38% of patients responded “Agree” over all items. “Disagree strongly” was the least chosen response category, for most items not exceeding 3% of answers. Mean response scores ranged from 3.1 (SD 0.9) for item 9 to 3.9 (SD 0.5) for item 1, see Table 2.

**Table 2:** PAM® item statistics (*n* = 554)

PAM® items	Order by IRT difference^a^	CTT^b^ difficulty	IRT difficulty	IRT discrimination	Infit mean squared	Infit *z*	Outfit mean squared	Outfit *z*
Item 01	1	3.9	− 2.9	0.9	1.1	0.4	0.9	− 0.5
Item 02	2	3.8	− 2.5	1.0	1.0	− 0.1	0.9	− 0.7
Item 03	3	3.7	− 2.3	0.8	1.0	0.0	0.9	− 1.0
Item 04	4	3.7	− 1.9	0.8	1.0	− 0.2	0.9	− 1.3
Item 05	7	3.4	− 1.2	1.0	0.9	− 1.4	0.9	− 2.2
Item 06	5	3.5	− 1.7	0.5	1.0	− 0.5	1.0	− 0.6
Item 07	10	3.3	− 1.0	0.7	0.9	− 1.4	0.9	− 1.9
Item 08	6	3.4	− 1.3	0.7	1.0	− 1.0	0.9	− 1.6
Item 09	13	3.1	− 0.5	0.9	0.9	− 2.4	0.9	− 2.7
Item 10	8	3.4	− 1.2	0.8	0.9	− 1.3	0.9	− 2.1
Item 11	9	3.4	− 1.2	1.1	0.9	− 1.5	0.8	− 2.7
Item 12	11	3.4	− 0.9	1.3	0.9	− 2.6	0.8	− 3.3
Item 13	12	3.3	− 0.9	0.8	0.9	− 1.6	0.9	− 2.0

^a^Item sequence by item response theory (IRT) difficulty, ordered from easiest to hardest

^b^*CTT* classical test theory. No missing data in PAM® items

Study participants showed a mean PAM® score of 74.1 (SD 13.7), see Table 3. 19 (3%) Were in patient activation level 1, 37 (7%) level 2, 91 (16%) level 3, and 407 (73%) level 4.

**Table 3:** Questionnaire scores

	Mean	Standard deviation	Possible range
PAM*®* score	74.1	13.7	1–100
Self-efficacy	3.3	0.5	1–4
Quality of life	4.5	0.9	1–6
General mood	4.5	0.7	1–6

*N* = 554

### Psychometric properties

Assumptions of IRT analysis were met sufficiently. The lowest two answer categories “Disagree strongly” and “Disagree” were chosen rarely, leading to estimation problems in the models. Therefore, these two categories were merged into one, named “Disagree strongly & Disagree”. Firstly, we estimated a GPCM with all available data. Estimations of the ability level on the latent trait were used to impute former “Not applicable” answers (*N* = 23, 0.3% of all values). In the final model, the latent ability patient activation was estimated by the answer patterns of the 13 items of 554 participants, estimating difficulty and discrimination of every single item as well. Fit indices indicated a good model fit: RMSEA 0.062 (95% CI 0.052–0.072), SRMSR 0.064, TLI 0.905, CFI 0.921, SABIC 11,496.6 and AIC_c_ 11,458.1. The patient activation ability score calculated from the GPCM correlated 0.98 (*p* < 0.001) with the PAM® score. Items 9 and 12 showed bad in- and outfit values. Items 5, 10, 11 and 13 showed bad outfit values. Item difficulties ranged between − 2.9 (item 1) to − 0.5 (item 9), see Table 2. Item discrimination ranged from 0.5 to 1.3, with a mean of 0.9 (SD 0.2). Item characteristic curves are displayed in Supplementary Fig. 1.

**Fig. 2:**
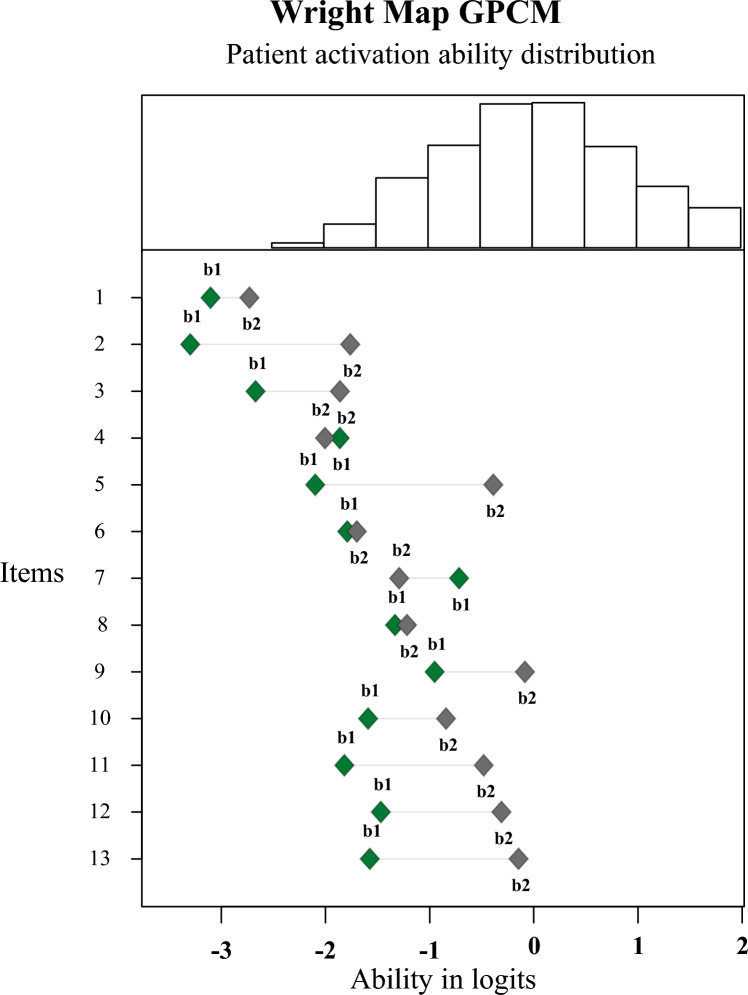
Wright map estimated by a GPCM. Top: distribution of the sample in activation scores in logits. Bottom: distributions of thresholds per item. The first threshold b1 describes the probability of choosing between “Disagree strongly & Disagree” or “Agree”, the second threshold b2 between “Agree” or “Agree strongly”

**Fig. 3:**
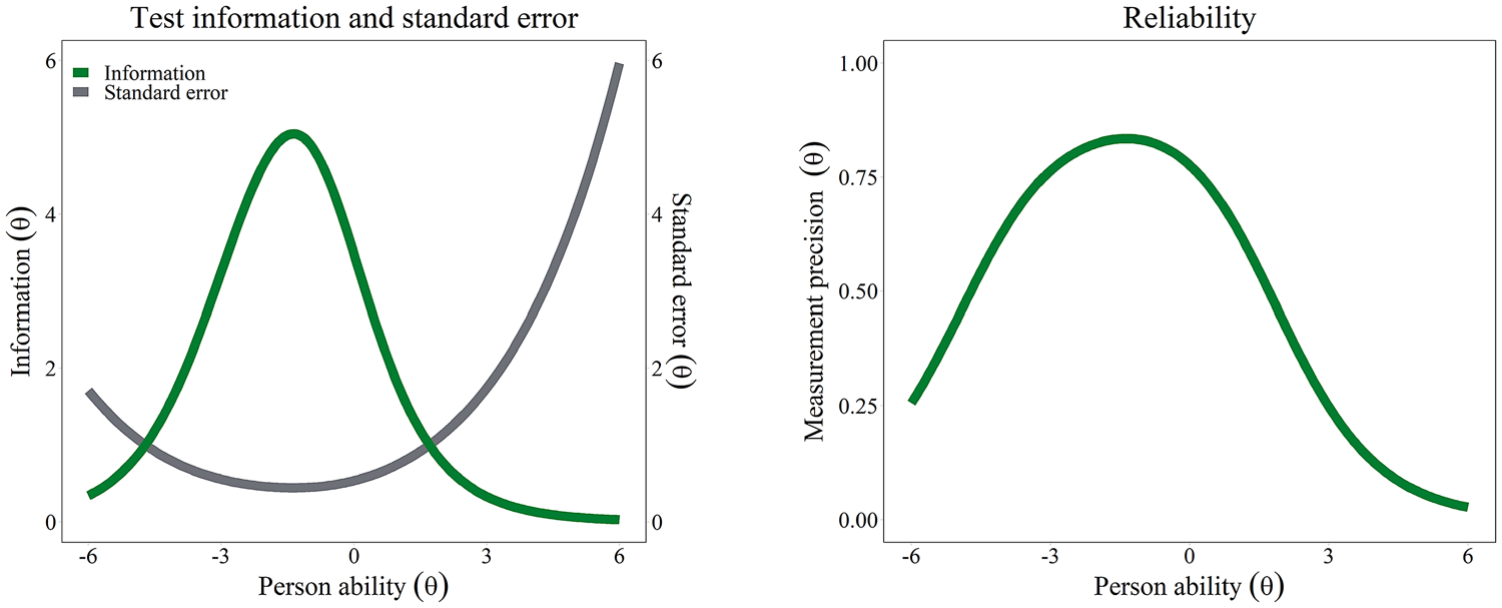
Left side: PAM® test information (green line) and standard error (grey line) over the ability range in logits. Right side: PAM® empirical reliability over the ability range in logits. (Color figure online)

Most of the sample had abilities between − 2 and 1 logits, see Fig. 2. There is no item with a threshold for an ability higher than 0 logits. Total test information of the PAM-13D is 22.6, see Fig. 3. Most information is given in the ability area of − 3 to 1 logits. Assessment of medium to highly activated patients is conflicted with more error than the lower ability range.

The GPCM estimated an empirical reliability of 0.75. Similarly, Cronbach’s *α* was 0.75, indicating acceptable reliability. Using the GPCM, the estimation revealed two empirically distinguishable groups by the PAM-13D (e.g., low and high activation).

DIF was found for interviewer VW compared to all other interviewers for item 7 (*p* < .001 *R*^2^ = 0.028, NCDIF = 0.123, Supplementary Fig. 2). This item was easier if administrated by interviewer VW.

PAM® score showed a Spearman correlation of − 0.34 (*p* < .001) with health status, 0.54 (*p* < .001) with self-efficacy, 0.51 (*p* < .001) with quality of life, and 0.34 (*p* < .001) with general mood. For comparison, the patient activation ability score calculated from the GPCM correlated − 0.35 (*p* < 0.001) with health status, 0.55 (*p* < .001) with self-efficacy, 0.52 (*p* < .001) with quality of life, and 0.34 (*p* < .001) with general mood.

## Discussion

This study shows that the read-aloud PAM® survey demonstrates adequate measurement precision and construct validity to screen for patient activation in an everyday clinical setting. Patients with lower and medium activation are better differentiated than those with high activation. Additional research is needed to examine concerns regarding the structural validity. Since it takes about 4 min to administer the PAM® survey in an interview setting, an integration in clinical routine seems feasible.

### Interviewer setting

Although these findings are promising, it must be considered that social desirability in an interviewer setting could influence the results. Participants could have over-reported their patient activation. As shown previously, compared to self-administration, interview responses are more positive and socially acceptable [20]. However, during the interview, most patients talked openly about their problems with self-care in their everyday life. Another aspect that could lead to systematically different scores is a potential risk of interviewers influencing patients' responses [21], particularly when item explanations are provided. Thus, it is crucial to prioritize interviewer training, which should include standardized explanations of items. This study specifically aimed to closely examine whether interviewers had an impact on patients' answer behavior. More patients agreed to item *7* when interviewed by VW. Although only a very small effect was observed, this shows that proficient interviewer training is essential for the validity of the results. Since an influence of the interviewer on the answer behavior was only found for one item, we conclude that an objective assessment in an interview setting using the PAM-13D is possible. Previously, in an interview setting with individuals with vision loss [22], results regarding the PAM® survey were comparable to those found in other samples without vision loss [19, 48]. In this study patients showed high patient activation, with a mean PAM® score of 74.1 (SD 13.7) and 73% of participants being in PAM® level 4. This score is higher than scores previously found in Europe [49]. A comparison of activation scores in Europe revealed the German speaking sample as the most activated one [49]. Recently in Germany, the PAM® survey was administered in an interview setting with multimorbid patients. Similar scores as in this study with a mean of 76.1 (SD ± 16.4) were reported for multimorbid patients [50]. Overall, these results indicate the feasibility of completing the questionnaire in an interview mode without being overly influenced by social desirability or interviewer bias.

Additionally, completing the PAM-13D in an interview setting created advantages: a conversation was partially developed and the interviewer got more information than the items asked for. Most of the patients answer more than just “Agree”—they do like to tell their story. For example, one patient had cookbooks for diabetics, but could not use recipes due to expensive ingredients. Actively managing her condition, she seeked guidance for preparing affordable, healthy meals. In a clinical setting, capturing additional information communicated by patients can aid in identifying the health challenges they are facing. A systematic review investigating the diagnosis of depression concluded that obtaining additional information from patients can aid in more specific management of health problems. The use of a self-administered questionnaire followed by an interview is recommended [51].

A further advantage of the interview setting was the opportunity for patients to inquire about the meanings of items they did not understand. Some items, especially item* 7*, required clarification through a standardized sentence.

Not only the interview setting, but also disease specific attributes may have an influence on patient activation. Many patients in this sample suffered not only from macular edema, but also diabetes or hypertension. The score of this study is higher compared to other samples with diabetes, where mean scores were 59 (SD ± 12) [19], 57 (SD ± 14) [52], 59 (SD ± 10) [18]; or patients suffering from hypertension 61 (SD ± 12) [53] and 61 (SD ± 16) or a sample having both, diabetes and hypertension 60 (SD ± 13) [54]. Therefore, differences in self-management tasks between different diseases must be considered when studying patient activation. Looking at severe chronic conditions, lung cancer patients showed similarly high activation scores as in this study [55]. Patients with life-threatening long-term illnesses (cardiac diseases or cancer) showed higher activation than other samples in the US [56]. Different vascular disease patient groups were investigated, the group with the most severe disease showed the most activation [17].

### The psychometric properties of the read-aloud PAM®

The read-aloud PAM® survey shows adequate psychometric properties. Our findings indicate moderate reliability (0.75). While the items precisely assess patient activation in the lower and medium ability range, measurements in the high ability range are more error-prone. The reliability found in this study is lower than the reliability PAM-13D validation study (0.84) [5] and other European translations in (Denmark: 0.89, Italy: 0.88) [14, 15]. This study's lower reliability may result from the highly activated sample and the lack of difficult items. Moreover, construct validity is supported by expected associations with external criteria: higher activation was associated with better self-rated health, higher self-efficacy, higher quality of life and better general mood [6, 7, 9, 52]. Diabetics exhibiting higher activation levels had a better health status [19].

Investigating PAM-13D items, “Not applicable” was chosen rarely, mostly for item 4 “I know what each of my prescribed medications does”; consistent with other studies. Not everyone with a chronic disease necessarily takes medication. For patients with a macular edema due to retinal vein occlusion, this can be their first diagnosis, without a history of a chronic disease yet.

In this study, all items showed ceiling effects, aligning with similar findings in other European countries [14, 15]. Consistent with previous studies, “Disagree strongly” was chosen in ≤ 10% of the responses [5, 15] necessitating merging with the “Disagree” category for analysis [8, 48].

During the analysis, six items showed a poor model fit, suggesting influences beyond patient activation on response behavior. These items *(5, 9, 10, 11, 12 and 13*) displayed highest difficulties in this study. To our knowledge, this was not found in other studies [6, 18, 22], but, they did not report the *z*-outfit, where the misfit occurred in our study.

According to the original questionnaire design, item difficulty increases sequentially, while items align with specific activation levels. This applied only to the first four items in this study. Similar results were found in the PAM-13D [5], other translations [14, 49] and a diabetic sample [18]. These findings, including ours, might result from specific disease and cultural factors. Although the original goal was to increase item difficulty over the course of the questionnaire, its absence in many studies is no problem for the interpretation of the overall PAM® score. Caution is advised when evaluating health behavior on a few items, as they may not measure the intended patient activation level. Moreover, the classification into four different activation levels could not be replicated in this study. Our analysis estimated how many groups the questionnaire distinguishes empirically. It suggested categorizing patients into two groups instead of four.

### Limitations and future recommendations

As in every survey, the question arises as to whether there is a sample bias. Uncertainty remains whether interviewed patients from one center are representative for patients with macular edema. It should be emphasized positively that almost all recurrent patients were interviewed during the study period of two years. A further concern is a possible selection bias. While this effect is described in many studies (e.g. [16]), selection bias in this study can only be small, since 94% of the potentially eligible patients could be included.

This study demonstrates administrating questionnaires to a population, unable to complete them by themselves, delivers reliable and valid results. It is possible to overcome barriers due to patients’ characteristics to gain more insight about patients’ health behavior. Therefore, in clinical practice and future research, an atmosphere should be established where the patient feels understood and his thoughts and feelings welcome to reduce social desirability. Since the interviewer situation has an influence on patients, a standardized procedure is necessary including standardized explanations for items.

### Conclusions

This study suggests the PAM® survey can be read-aloud in everyday clinical settings to assess patient activation of patients with chronic diseases. All items showed ceiling effects and these were rarely disagreed. This raises the question of whether it is necessary for all items to be answered with four response categories. Due to the interview setting, we recognized that some items are not well understood. Proficient interviewer training is essential to ensure consistent explanation for patients. While the PAM® survey effectively captures activation in lower to middle-activated patients, it falls short for highly activated patients. Therefore, we recommend using it as a screening tool rather than a diagnostic measure. To enhance the ability to discern highly activated patients and better understand the challenges they face, we propose rephrasing existing items to increase difficulty or incorporating new, specific items. Despite limitations, PAM® survey is valuable in identifying low activation, creating the opportunity to encourage skills, knowledge, and confidence in the management of a chronic disease.

### Supplementary Information

Below is the link to the electronic supplementary material.

Supplementary file1 (DOCX 79 kb)

## Data Availability

The participants of this study did not give written consent for their data to be shared publicly, so due to the sensitive nature of the research the data can not be made available.

## Abbreviations

AIC_c_: Akaike information criterion corrected
BMI: Body mass index
CFI: Comparative fit index
CTT: Classical test theory
DIF: Differential item functioning
GPCM: General partial credit model
IRT: Item response theory
NCDIF: Non-compensatory differential item functioning
RMSEA: Root mean square error of approximation
SABIC: Sample adjusted Bayesian information criterion
SRMSR: Standardized root mean square residual
TLI: Tucker–Lewis index
PAM®: Patient activation measure
