# Supplementary material for: Measuring patient activation: the utility of the Patient Activation Measure administered in an interview setting
Source: Qual Life Res. 2024 Feb 22;33(5):1389–400. doi: 10.1007/s11136-024-03614-2 (PMC11045573; doi:10.1007/s11136-024-03614-2)
Supplement: Supplementary file 1 — Supplementary file1 (DOCX 79 kb) [file 11136_2024_3614_MOESM1_ESM.docx]

# Supplementary

# Sample size

The primary aim of this study is to analyze the PAM® survey using a suitable IRT model. Sample size requirements are dependent upon the complexity of the chosen IRT model, with increased model complexity necessitating a larger sample size. Moreover, a greater number of participants is needed when the questionnaire provides more response options, as it requires estimation of additional item parameters [1]. A minimum sample size of 500 is suggested for a two-parameter model, with the goal of obtaining precise and stable estimates for item characteristics and individual scores [2]. Furthermore, the power of a Rasch model can be computed through differential item functioning (DIF), assuming the existence of a criterion that divides participants into at least two groups [3]. Simulations indicate that as the number of participants increases and the number of items decreases, the ability to detect DIF improves. Given that the PAM® survey consists of 13 items with 4 response options, and considering the estimation of item characteristics and person scores, the target was to include at least 500 patients. To adequately assess various subgroups and potential DIF, it was intended to include a larger sample size (N=700) in this study. The initial plan to include 700 patients in one year appeared feasible, considering the annual inflow of approximately 1200 patients with newly diagnosed macular edema caused by diabetes or retinal vein occlusion in the outpatient clinic in Graz.

1. Cappelleri JC, Lundy JJ, Hays RD. Overview of classical test theory and item response theory for the quantitative assessment of items in developing patient-reported outcomes measures. Clin Ther. 2014;36:648–62.

2. Reeve BB, Fayers P. Applying item response theory modeling for evaluating questionnaire item and scale properties. Assess Qual Life Clin Trials Methods Pract. 2005;2 Journal Article:55–73.

3. Kubinger KD, Rasch D, Yanagida T. On designing data-sampling for Rasch model calibrating an achievement test. Psychol Test Assess Model. 2009;51:370.

Supplemental table 1. Item fit statistics for different polytomous IRT models estimated with three response categories before imputation.

|  | RMSEA | 95% Confidence Interval | SRMSR | TLI | CFI | SABIC | AICc |
| --- | --- | --- | --- | --- | --- | --- | --- |
| Rating scale model | 0.069 | 0.060 -0.077 |  | 0.882 | 0.866 | 11488.08 | 11504.3 |
| Partial credit model | 0.062 | 0.053 - 0.072 | 0.075 | 0.903 | 0.904 | 11439.66 | 11467.6 |
| GPCM | 0.058 | 0.048 - 0.068 | 0.064 | 0.915 | 0.929 | 11431.14 | 11469.6 |
| Graded response model | 0.066 | 0.056 - 0.076 | 0.063 | 0.891 | 0.910 | 11399.22 | 11437.7 |
| RMSEA=root mean square error of approximation, absolute fit index, difference between hypothesized model and perfect model. SRMSR= standardized root mean square residual, absolute fit index, average standardized residual covariance, under the fitted model. TLI=Tucker-Lewis index, CFI=comparative fit index, incremental fit indices difference between hypothesized model and baseline model (model with the worst fit). SABIC=sample adjusted Bayesian information criterion. AICc = Akaike information criterion corrected; relative model fit indices, smaller is better, for comparing fit among two or more models. | | | | | | | |


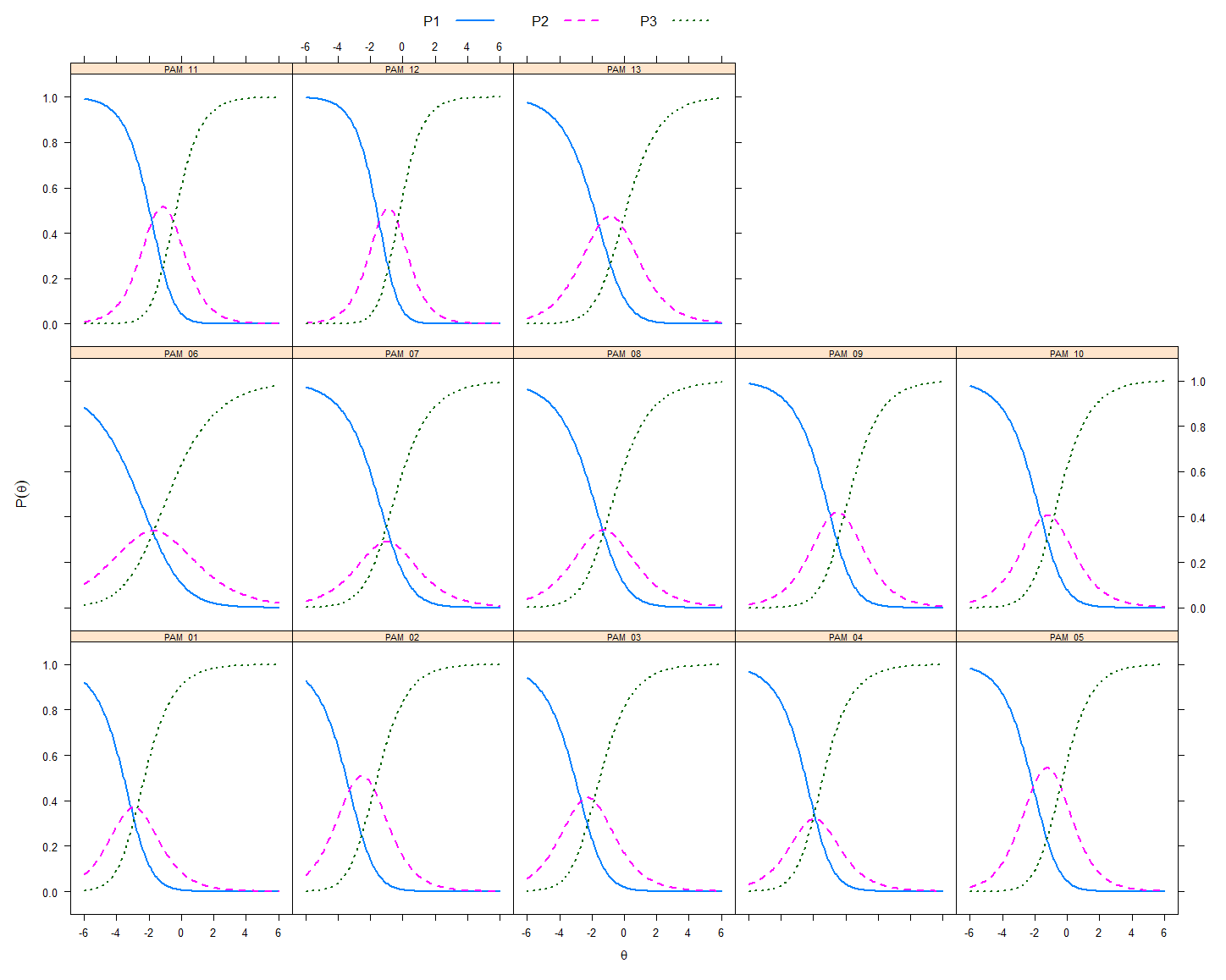


Supplemental. Figure 1. PAM® item characteristic curves estimated by a GPCM with three answer categories. Probability (0 to 1) for choosing a specific answer category over the whole ability range displayed in logits (-6 to 6). P1 = “Disagree strongly & Disagree”; P2 = “Agree”, P3 = “Agree strongly”.


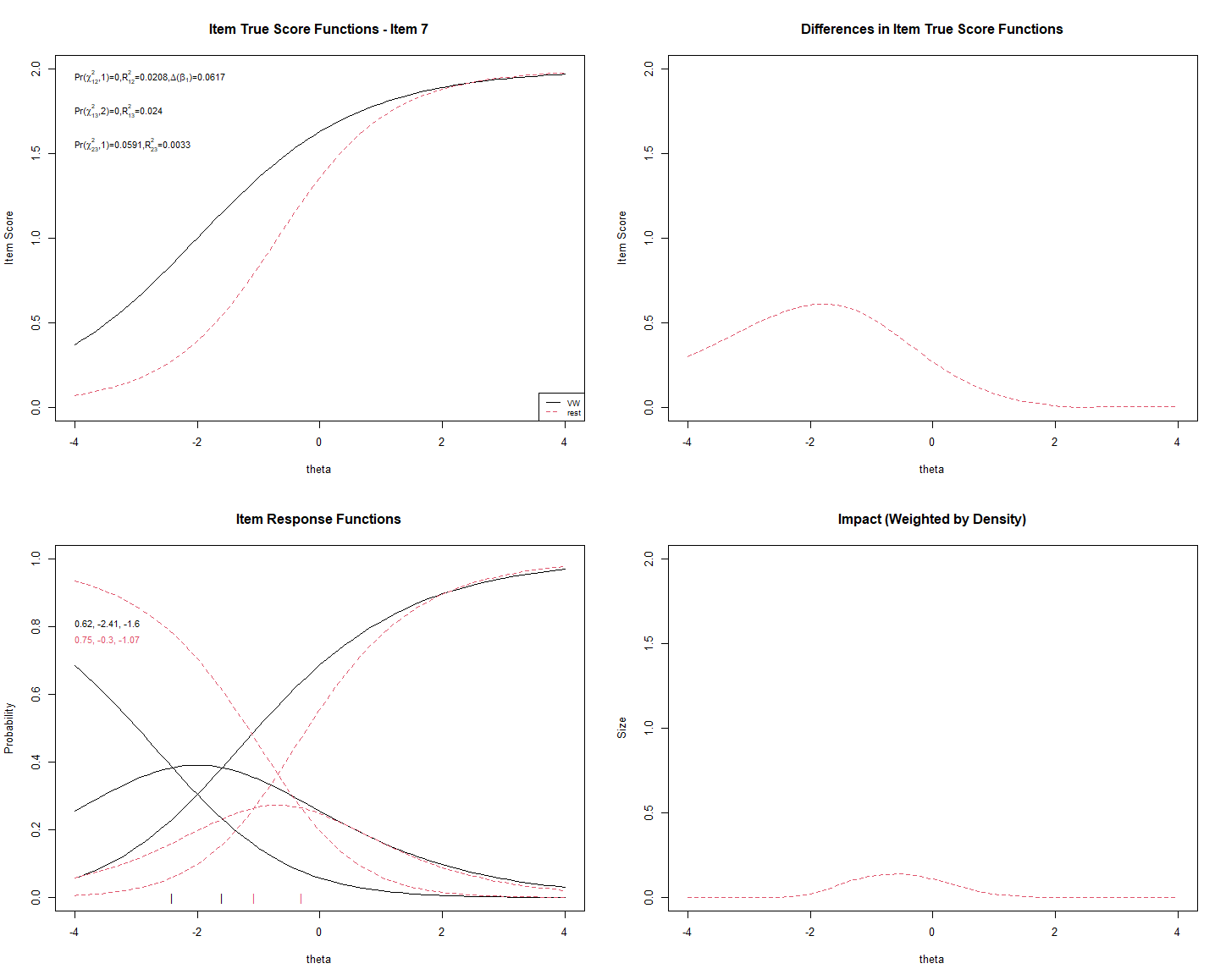


Supplemental. Figure 2. Item 7: Uniform DIF for interviewer 2 compared to the other interviewers, presented as differences in item true score functions, displayed as the dotted line. Theta = person ability in logits.

# R-Code used for analysis

# Load packages

library(mirt)
library(lavaan)
library(psych)
library(psychTools)
library(tidyverse)
library(flextable)
library(crosstable)
library(WrightMap)
library(knitr)

# Load data

load("C:/xxx.RData")

#Select sample for main analysis
filter <- act %>% select(ID, HS_01, FSOZU, SWE, HSWBS) %>% na.omit() %>% select(ID)
act <- merge(filter, act, by="ID")

data <- select(act, starts_with(c("PAM_")))

#Merge lower two response categories due to low cell occupation
data2 <- data
data2[data2 == 1] <- 2

# 1. assumption: unidimensionality

## CFA with three categories

invisible(mod1 <- (mirt(data2, 1)))
mod1s <-(summary(mod1))
mod1f <- cbind(mod1s$rotF, mod1s$h2)
kable(mod1f, digits=2)

# Final model: generalized partial credit model

## Model calculation

results.gpcm <- mirt(data=data2, model=1, itemtype="gpcm", SE=TRUE, verbose=FALSE)
show(results.gpcm)

## Fit indizes

fit1.gpcm <- M2(results.gpcm, type='C2', na.rm=TRUE)
AICc <- extract.mirt(results.gpcm, 'AICc')
SABIC <- extract.mirt(results.gpcm, 'SABIC')
HQ <- extract.mirt(results.gpcm, 'HQ')
fit2.gpcm <- cbind(AICc,SABIC, HQ)

fit.gpcm <- cbind(fit1.gpcm, fit2.gpcm)
rownames(fit.gpcm) <- c("GPCM")
kable(fit.gpcm, digits=c(rep(3,9),rep(1,3)))

## Empirical plots

itemfit(results.gpcm, empirical.plot = 1, na.rm=TRUE)
itemfit(results.gpcm, empirical.plot = 2, na.rm=TRUE)
itemfit(results.gpcm, empirical.plot = 3, na.rm=TRUE)
itemfit(results.gpcm, empirical.plot = 4, na.rm=TRUE)
itemfit(results.gpcm, empirical.plot = 5, na.rm=TRUE)
itemfit(results.gpcm, empirical.plot = 6, na.rm=TRUE)
itemfit(results.gpcm, empirical.plot = 7, na.rm=TRUE)
itemfit(results.gpcm, empirical.plot = 8, na.rm=TRUE)
itemfit(results.gpcm, empirical.plot = 9, na.rm=TRUE)
itemfit(results.gpcm, empirical.plot = 10, na.rm=TRUE)
itemfit(results.gpcm, empirical.plot = 11, na.rm=TRUE)
itemfit(results.gpcm, empirical.plot = 12, na.rm=TRUE)
itemfit(results.gpcm, empirical.plot = 13, na.rm=TRUE)

## Item statistics

cat("Itemfit statistics")
kable(itemfit(results.gpcm, fit_stats = c("S_X2","infit"), na.rm = TRUE), digits=2)
cat("Item difficulty parameter")
kable(gen.difficulty(results.gpcm, type = "IRF"), digits=2)
cat("Discrimination parameter")
kable(MDISC(results.gpcm), digits=2)

## Item characteristic curves

#Option characteristic curves
plot(results.gpcm, type = 'trace',
 main = "", par.settings = simpleTheme(lty=1:4,lwd=2),
 auto.key=list(points=FALSE,lines=TRUE, columns=4), theta_lim = c(-6, 6))

#Item information function
plot(results.gpcm, type = 'infotrace',
 main = "", par.settings = simpleTheme(lwd=2, col="#007934", font="serif") , theta_lim = c(-6, 6))

## Test information

plt <- plot(results.gpcm, type = 'infoSE') #store the object
pltdata <- data.frame(lapply(plt$panel.args, function(x) do.call(cbind, x))[[1]])
#Estimate standard error
pltdata$yse<- 1/(sqrt(pltdata$y))

#Plot
ggplot(pltdata, aes(x)) +
 geom_line(aes(y = yse, colour = "#bfc0c2"), lwd=4) + geom_line(aes(y = y, colour = "#007934"), lwd=4) +
 scale_y_continuous(expression(paste('Information '(theta)), size=44),
 sec.axis = dup_axis(name=expression(paste('Standard error '(theta)), size=44))) +
 xlab(expression(paste('Person ability '(theta)), size=44)) +
 theme_light() +
 theme(panel.grid.minor = element_blank(), panel.grid.major = element_blank(), panel.border=element_rect(color="black", fill=NA),
 plot.title = element_text(hjust = 0.5, size=48), text=element_text(size=44, family="serif"),
 axis.text.x = element_text(color="black", size=40), axis.text.y = element_text(color="black", size=40), legend.position = "bottom", legend.title=element_blank())+
 scale_colour_manual(values =c('#007934'='#007934','#bfc0c2'='#bfc0c2'), labels = c('Information','Standard error'), name=NULL)

## Wright Map

Theta <- fscores(results.gpcm)
coefG <- coef(results.gpcm, IRTpars = TRUE, simplify = TRUE)
Thresh <- coefG[["items"]]

Thresh <- as.data.frame(Thresh[ ,-1])
#Rename items
rownames(Thresh) <- c("1", "2", "3", "4", "5", "6", "7"," 8", "9", "10", "11", "12", "13")

par(family="serif",ps = 18)

wrightMap(thetas=Theta, thresholds = Thresh, vertLines = TRUE,
 thr.sym.col.bg =rep(c("#007934", "#6b6d74"), each=13), thr.sym.cex = 1.4,
 main.title = "Wright Map GPCM", axis.persons = "Patient activation ability distribution", dim.names = " ", family = "A")

## Correlation plot

act2 <- data.frame(act, Theta)

par(family="serif",ps = 19)
act2 %>% select(PAM, F1) %>% pairs.panels(stars=FALSE, method="spearman", hist.col="#007934", rug = FALSE, cex.cor=1, digits = 2, breaks = 50, smooth=FALSE, ellipses = FALSE)

## Reliability

theta_se <- fscores(results.gpcm, full.scores.SE = TRUE)
cat("Empirical")
print(empirical_rxx(theta_se))

plot(results.gpcm, type="rxx", lwd=2)

cat("Marginal")
print(marginal_rxx(results.gpcm))

## Person reliability and person separation index

#To calculate the MSE, I need the SE of my model
## Get the SE from my fitted model
sedata <- as.data.frame(fscores(results.gpcm, full.scores.SE = TRUE))
MSEp <- sum(sedata$SE_F1^2) #141.9957

#SSDp
meanp <- mean(act2$F1)
act2$diffpc <- meanp - act2$F1
SSDp <- sum(act2$diffpc^2) #411.9926

#Person reliability index
pri <- 1 - (MSEp/SSDp) #0.655
cat("Person Reliability Index")
print(pri)

#Separation

G <- sqrt((pri/(1-pri)))
cat("Person Separation Index")
print(G)

cat("Count of groups empirical")
#Count of groups H = (4G + 1)/3
nstrata <- (4*G+1)/3
print(nstrata)

# Cronbach’s alpha

pam13 <- list(PAM= c("PAM_01", "PAM_02", "PAM_03", "PAM_04", "PAM_05", "PAM_06", "PAM_07", "PAM_08", "PAM_09", "PAM_10", "PAM_11", "PAM_12","PAM_13"))
pam <- scoreItems(pam13, data2)

cat("Alpha")
kable(pam$alpha, digits=3)
cat("Scale intercorrelations corrected for attenuation raw correlations below the diagonal, alpha on the diagonal corrected correlations above the diagonal")
kable(pam$corrected, digits=3)
cat("Average item correlation")
kable(pam$av.r, digits=3)
cat("Median item correlation")
kable(pam$med.r, digits=3)
cat("Item by scale correlations: corrected for item overlap and scale reliability")
kable(pam$item.cor,digits=3)

# Trait-trait correlations

cat("With overall scores")
act %>% select(PAM, FSOZU, HS_01, HSWBS_GS, HSWBS_LZ, SWE) %>% pairs.panels(stars=TRUE, method="spearman", hist.col="#007934", rug = FALSE, cex.cor=1, digits = 2, breaks = 50, smooth=FALSE, ellipses = FALSE)
cat("Signif. codes: '***' 0.001 '**' 0.01 '*' 0.05")
act %>% select(PAM, FSOZU, HS_01, HSWBS, SWE) %>%corr.test(method="spearman") %>% print(short=FALSE) %>% kable()
